# An algorithm for Parkinson’s disease speech classification based on isolated words analysis

**DOI:** 10.1007/s13755-021-00162-8

**Published:** 2021-07-30

**Authors:** Federica Amato, Luigi Borzì, Gabriella Olmo, Juan Rafael Orozco-Arroyave

**Affiliations:** 1 Department of Control and Computing Engineering, Politecnico di Torino, Corso Duca degli Abruzzi 24, Turin, Italy; 2 GITA Lab, Faculty of Engineering, University of Antioquia, Medellín, Colombia; 3 Pattern Recognition Lab., Friedrich-Alexander-Universit at Erlangen-Nu rnberg, Martenstrasse 3, Erlangen, Germany

**Keywords:** Parkinson’s disease, Speech impairment, Speech analysis, Isolated words, k-Nearest neighbours, Artificial Intelligence, Telemedicine

## Abstract

**Introduction:**

Automatic assessment of speech impairment is a cutting edge topic in Parkinson’s disease (PD). Language disorders are known to occur several years earlier than typical motor symptoms, thus speech analysis may contribute to the early diagnosis of the disease. Moreover, the remote monitoring of dysphonia could allow achieving an effective follow-up of PD clinical condition, possibly performed in the home environment.

**Methods:**

In this work, we performed a multi-level analysis, progressively combining features extracted from the entire signal, the voiced segments, and the on-set/off-set regions, leading to a total number of 126 features. Furthermore, we compared the performance of early and late feature fusion schemes, aiming to identify the best model configuration and taking advantage of having 25 isolated words pronounced by each subject. We employed data from the PC-GITA database (50 healthy controls and 50 PD patients) for validation and testing.

**Results:**

We implemented an optimized k-Nearest Neighbours model for the binary classification of PD patients versus healthy controls. We achieved an accuracy of 99.4% in 10-fold cross-validation and 94.3% in testing on the PC-GITA database (average value of male and female subjects).

**Conclusion:**

The promising performance yielded by our model confirms the feasibility of automatic assessment of PD using voice recordings. Moreover, a post-hoc analysis of the most relevant features discloses the option of voice processing using a simple smartphone application.

## Introduction

Parkinson’s disease (PD) is a chronic and progressive neurodegenerative disorder, affecting about 1% of individuals over the age of 60 [40]. According to several epidemiological studies conducted both in Europe and in the USA, PD affects the male population approximately 1.5 times more than the female population [28]. Following the disease onset, PD patients face progressive disability, with significant impact on the activities of daily living. Both motor and non-motor symptoms are consequent to the degeneration of dopamine neurons, which occurs especially in the substantia nigra pars compacta region of the midbrain [28].

The cardinal motor symptoms of PD include rigidity, tremor at rest, bradykinesia (i.e. slowness in movement execution), and postural instability. As a result, a reduction in the quality of life and an increase in the risk of falls in the PD population are observed [15, 20].

On the other hand, non-motor symptoms include olfactory impairment, orthostatic hypotension, constipation, sleep disturbances, and speech impairment. Behavioral problems, depression, and anxiety frequently occur, and dementia is quite common in the advanced stages of the disease [21]. Parkinson’s disease diagnosis is currently based on a detailed neurological examination, inclusive of a review of the patient medical history and a clinical evaluation of motor and non-motor symptoms and supported by the dopamine transporter (DAT) scan, if need be [28].

Monitoring of the disease progression is generally performed a few times a year during outpatients appointments. At present, MDS-UPDRS (Movement Disorder Society revision of the Unified Parkinson’s Disease Rating Scale) is universally employed to assess the course of PD after its diagnosis [20]. It involves four parts related to non-motor experiences of daily living, motor experiences of daily living, motor examination, and motor complications, respectively. Disease staging is entrusted to the clinician’s expertise; hence, it is sometimes considered excessively operator-dependent, in particular in early-stage PD or during the assessment of specific pathological conditions. Therefore, technological research on PD focuses on developing tools for diagnostic support and continuous follow-up, through the analysis of biomedical signals correlated to patient conditions.

In this context, speech impairment carries significant information and plays a pivotal role in the early detection and follow-up of the disease. It is well known that PD patients encounter loss of prosody and clarity due to dysfunctions in the different systems involved in speech production. According to [7], alterations in voice and speech occur in approximately 75–90% of the PD population, with voice and prosody being the earliest indicators of PD [4, 18, 41]. In more detail, dysarthria is a neuro-motor disorder involving the motor component of the speech production process and it is related to respiratory limitations, reduced elongation or adduction of vocal cords, and disturbances at the articulatory level. It is characterized by poor articulation of the phonemes (alterations in force, speed, volume, tone, range, or precision of movements necessary for voice control [25]), yet intact language understanding and ideation. The typical neurological signs evaluated on PD patients encompass reduced loudness and pitch variability, breathy or hoarse voice, imprecise articulation, and more general features such as abnormalities of speech rate and pause ratio [31]. The symptoms become more pronounced as the disease worsens.

From an engineering perspective, the human vocal signal can be seen as a quasi-periodic train of air pulses that are shaped by the resonances of the vocal tract [27]. The frequency of the train pulses, i.e. the number of glottal contraction per second, represents the fundamental frequency (*F*0) or *pitch*, while the resonance frequencies of the oropharyngeal cavities account for the *vocal formants*. *F*0 is influenced by the intrinsic features of the phonatory system and is distinctive of the single speaker to a large extent. It is also influenced by anatomical characteristics dependent on the speaker’s gender. In fact, although values may differ according to the language taken into account, the mean *F*0 value for healthy male population is 120 Hz, while in female subjects it reaches 220 Hz [38].

Automatic methodologies for PD voice analysis mainly make use of sustained vowel phonation tests. Since the set of extracted features is task-dependent and the possibility of achieving sounds information from word-repetition databases is still under investigation, a validated and interpretable features set for this specific task has not been defined up to now. However, besides being easy, fast, and not depending upon the patient’s ability to interpret detailed instructions, the analysis of isolated words could be more effective than sustained phonation in assessing PD dysarthria. It is our belief that the pronunciation of many different words represents a valuable source of information about the patient’s condition and the staging of diseases. Moreover, the neurologist or the speech and language therapist could arrange particular sets of words to evaluate specific aspects of movement and articulation control.

The first objective of this work is to define and validate a set of features suitable for analyzing recordings of PD patients pronouncing isolated words. The second objective is to devise analysis tools that are somewhat demanding in terms of processing capabilities and data quality. This could enable both voice recording and data analysis on a simple platform such as a smartphone, as also proposed in [23]. We are confident that easy-to-use and inexpensive tools can actually help in the patient’s follow-up at home, and can even be *prescribed* by neurologists, similarly to drugs.

The remainder of this paper is organized as follows: in section “Related work” we review recent automatic methodologies for PD patients speech analysis; in section “Materials and methods” we describe the employed dataset, the feature extraction and selection methods, and the classification model. In section “Results and discussion” we address classification performance and statistical analysis findings; finally, in section “Conclusions and future work” we draw conclusions and propose further improvements for the present algorithm.

## Related work

There are several studies focusing on PD speech analysis, including different recording tasks. Sustained vowels phonation is perhaps the most popular, also because it represents a very common task in different applications [24]. Other works focus on continuous speech recordings including sentences, read texts, and spontaneous speech, where clinically informative phenomena like prosody can be analyzed [19, 37, 48]. Few papers focused on the production of isolated words. One of the earliest studies addressing this task in PD patients is [36]. This work investigated the discriminant capability of spectral and cepstral features extracted from a set of 24 isolated words and 5 vowels pronounced by Colombian Spanish speakers. The authors performed the classification between controls and people with PD employing a support vector machine (SVM) with Gaussian kernel and compared the results achieved with each set of features separately and with the fusion of all coefficients. Accuracy of 92% and 79% for words and vowels respectively, was achieved when all utterances and features were merged into the same representation space. Despite satisfactory results, the methodology was very basic and no pre-processing was applied.

In [37] the authors included isolated words among other speech tasks. The employed database included native speakers from Spain (50 healthy controls-HC, 50 PD), Germany (88 PD, 88 HC), and Czech Republic (20 PD, 16 HC). The work addressed the automatic classification of HC and PD speakers and compared the results achievable with different languages and sets of features. Utterances in each corpus were modeled with four sets of parameters, in order to detect different facets of the language impairment. The approaches included: (1) modeling of irregular movements of the vocal tract based on Mel-Frequency Cepstral Coefficients-Gaussian Mixture Models (MFCC-GMM) super vectors; (2) prosody analysis by means of F0, energy, duration, and pauses; (3) characterization of voiced frames through noise content, formants, and MFCCs; (4) analysis of energy in unvoiced frames using MFCCs and Bark Band Energies (BBE). Based on their results, the authors stated the robustness of the latter approach, which led to classification accuracy ranging from 85 to 99% using a radial basis SVM.

Both the mentioned works exploited the PC-GITA database, but none of these investigated the generalization capability of models on a separate and independent dataset. Additionally, the models proposed in [37] were optimized in test, and this yielded too optimistic results, as also stated by the authors themselves.

More recently [22, 49] addressed the same corpus as in [37]. More in detail, Zahid et al. [49] proposed three methods based on transfer learning, deep feature extraction, and classic machine learning, respectively. Although the results achieved for other tasks were very satisfactory, the highest accuracy reported for isolated words, employing the transfer-learning approach, was 77%. In [22], the authors proposed a method for isolated words modeling based on features extracted from the Hilbert Spectrum to characterize non-linearities and non-stationarities of the speech signal. The performance of the employed classifier (SVM with Gaussian kernel) showed that the coefficients proposed, namely Instantaneous Energy Deviation Coefficients (IEDCC), outperform the classical acoustic features, achieving accuracy ranging from 81 to 91% when addressing isolated individuals words. The authors did not present the results of merged features, but they used an additional test set encompassing 20 PD patients and 20 healthy controls; the best-reported accuracy was 82%.

In the present work, we undertake a methodology based on different signal processing and pattern recognition techniques applied to the analysis of isolated words. First, we implemented a pre-processing step, which was followed by a multi-level feature extraction procedure and a classification step. The main contributions of the paper include: the multi-level feature extraction approach, which allows deriving multiple and specific facets of vocal alteration; the introduction of new features to characterize the voice impairment of PD patients; the use of a separate and independent test set, which allows for more general and realistic results.

## Materials and methods

This section describes the datasets employed and the algorithm developed for the classification of PD patients’ voices.

In this work, we carried out a multilevel analysis to assess the level of detail necessary to achieve the best trade-off between complexity and classification accuracy. Starting from a set of high-level parameters extracted from the non-segmented signal, we progressively added features derived first from the voiced regions and then from the transition regions. This approach allowed a detailed analysis of the speech impairment in PD patients. In fact, voiced segments bear information about the harmonic component of the signal, while transition zones, which describe the passage from voiced to unvoiced regions and vice-versa, are assumed to model the loss of motor control and the difficulty to start and stop movements typical of PD patients. Finally, features extracted from the non-segmented signal are representative of the overall sound. Following this approach, we extracted a total number of 126 features from the entire signal, voiced segments, and on-set/off-set regions [35, 37]. Moreover, since the employed datasets encompass 25 isolated words spoken by each subject, we analyzed the possibility of obtaining better classification results by combining the features extracted from different words into the same representation space.

It is worth emphasizing that we have devoted considerably higher efforts to feature extraction and selection than to classification itself. This choice is in line with the objectives of our work, i.e. to obtain a well-assessed, lightweight, simple and fast model that can be used for on-device analysis (e.g. smartphone applications).

More in detail, we performed a robust pre-processing described in section “Pre-processing”; we extracted a large number of features, both acknowledged and not hitherto used for PD detection, as discussed in section “Feature extraction”; we performed a hard feature selection, reported in section “Feature selection”; finally we performed classification as described in section “Classification”.

**Fig. 1:**
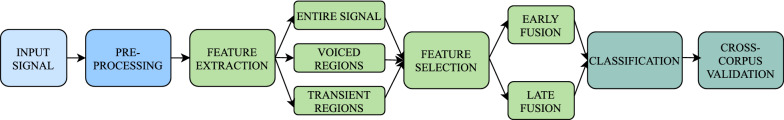
Work flow scheme

Figure 1 depicts a simple flowchart to provide a general overview of the workflow.

### Dataset

#### PC-GITA dataset

The main database used in this study is the PC-GITA, a well balanced corpus in terms of age and gender that includes 100 Colombian Spanish speakers [33]. More in detail, it encompasses 50 patients with PD and 50 HC (50% male and 50% female). The age of the male PD population ranges from 33 to 77 years old ($62.2 \pm 11.2$), while for the female population it ranges from 44 to 75 years old ($60.1 \pm 7.8$). For the HC group, the ages of men and women range from 31 to 86 ($61.2 \pm 11.3$) and from 43 to 76 years old ($60.7 \pm 7.7$), respectively.

All voice samples were recorded with the patients in ON-state, i.e. no more than 3 h after the morning medication. None of the HC subjects had symptoms associated with PD or any other neurological disease.

Speech samples were captured under controlled noise conditions and with a professional audio setting (professional microphone and a Fast Track C400 sound card). The sample rate is 44.1 kHz with a 12-bit resolution. The speech task considered in this study is the repetition of 25 Spanish isolated words.

The recording of the PC-GITA corpus was carried out in accordance with the Declaration of Helsinki and it was approved by the Ethical Research Committee of Antioquia University’s Faculty of Medicine [33].

#### Additional dataset

Since PC-GITA samples were recorded under optimal recording conditions that are difficult to reproduce in real-life situations, we decided to include in this study another database to run cross-corpus validation and verify the results in a more realistic scenario. This second database includes 18 Spanish PD patients and 19 Spanish HC (46% male and 54% female). The age of the male PD population ranges from 54 to 78 years old, while in the female PD group it ranges from 50 to 83 years old. As for HC, men were aged 41 to 78, while women 29 to 78 years. The samples belonging to this second corpus were recorded in a quiet room with regular headsets. The two databases include the same set of 25 words and have been approved by the same ethics committee, with the only difference that, while the PC-GITA is a widely used public database, the second is currently private. The recordings of this additional database were captured at a sampling frequency of 16 kHz with a 16-bit resolution. The two datasets are characterized by different sampling rates; hence, all recordings were down-sampled to 16 kHz to maintain similar spectral conditions.

**Fig. 2:**
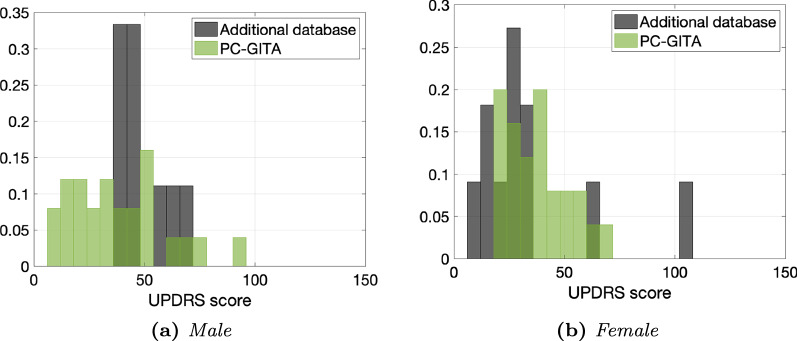
UPDRS distribution comparison between PC-GITA and the additional dataset for male and female subjects

Since most of the features extracted from vocal signals are influenced by the gender of the speaker, we split each dataset into two groups, based on the speaker’s gender. Then, we applied the same workflow to each cluster. Figure 2 shows the UPDRS total scores distribution for the PD patients included in the two corpora.

All of the participants in this corpus and in PC-GITA signed an informed consent which was revised and approved by the Ethical Committee of the Research Institute in the Faculty of Medicine at the University of Antioquia (approval 19-63-673). Further details of the two datasets can be found in [22, 33].

### Pre-processing

This section describes the pre-processing steps carried out to ease the extraction of specific information from vocal signals. This was performed through six different stages, described in the following. It is worth noting that the visual and acoustic signal examination indicated the absence of initial or final silence regions; hence no further preparatory steps were required.

#### Denoising

Signals were low-pass filtered to reduce distortion and background noise. To minimize the phase distortion in the pass-band, a 10-order zero-lag Butterworth low pass filter was employed, with cutoff frequency $3750\,Hz$, as also suggested in [11].

#### Normalization

The signals amplitude was normalized in the range $[-1, 1]$ to prevent the speaker-microphone distance from affecting the model.

#### Detrending

Detrending is necessary to remove slow fluctuations of the signal, which have no physiological significance but only hinge upon the recording system. This step is of crucial importance in eluding errors during the feature extraction task.

#### Segmentation

In order to perform the analysis of segments generated during vocal folds vibration, we employed the Praat software to detect start- and end-points within voiced regions. Moreover, as highlighted in [34], PD patients exhibit difficulties in producing *plosives*, which are transient-type sounds made up by abruptly releasing the airflow that has been previously blocked [2]. Therefore, after detecting voiced delimiters, we identified 160 ms windows centered on the edge of each chunk. According to [47], this window size allows to perform an in-depth analysis of the transient regions.

#### Framing

Vocal signals exhibit non-linear and complex behavior, which cannot be identified with the simple extraction of features from the entire recording epoch. The short-time analysis is usually employed to overcome this problem: each signal is divided into frames, which can reasonably be assumed to be stationary or quasi-stationary. According to [14], a frame size in the range 20–40 ms is usually considered to ensure two to three pitch periods within a frame, while maintaining the quasi-stationary assumption. In more detail, the length of the window is set according to the analysis to be performed and to the speech task. A common value employed for isolated words is 20 ms [22, 29].

#### Windowing

When performing the framing procedure, attention must be paid to the raise of a discontinuity in the area between two consecutive chunks, as this would lead to frequency distortion. Therefore, it is common practice to multiply each frame with a Hamming window [14] prior to spectral analysis. This process is described in Eq. 1, where N stands for the total number of samples.

1 $$\begin{aligned} w(n) = 0.54 - 0.46 \cdot \cos \left( 2\pi \frac{n}{N} \right) , \quad 0 \le n \le N -1 \end{aligned}$$

Overlap regions ranging from 0 to 75% are usually applied [17] to avoid the loss of information (i.e. signal attenuation) generated by the intrinsic structure of the Hamming window. In our specific application we set an overlap window equal to 50% of the window length, also in accordance with [14, 22, 29, 49].

### Feature extraction

Raw vocal signals do not provide much information unless a proper feature extraction procedure is implemented. In this work, we performed a multi-level analysis by combining a total number of 126 features extracted from the entire signal, voiced segments, and on-set/off-set regions.

**Table 1:** Overview of the extracted features, divided according to the domain of analysis

Region	Study	Feature	Information
Entire signal	[22]	IEDCC(1–6)	Vocal tract and vocal folds abnormalities [22]
	[3, 10, 12]	Zero crossing rate$^{H}$	Voice activity (Details in [1])
	[10, 16, 22]	DFA$^{H}$	Self-similarity of the voice (Details in [14])
Voiced	[16]	Bandwidth$^{L}$	Frequency range
	[10, 16, 43]	Harmonic ratio$^{L}$	Ratio of signal over noise [16]
	[10, 16, 43]	F0$^{L}$	Vocal folds vibration and frequency alteration
	[46]	Spectral features: flux$^{L}$, skewness$^{L}$, entropy$^{L}$, crest$^{L}$, flatness$^{L}$, slope$^{L}$, roll off$^{L}$, spread$^{L}$, centroid$^{L}$, kurtosis$^{L}$	Spectrum shape information (Details in [1])
	[6]	LPC(1–3)$^{L}$	Formants and resonances (Details in [1])
	[10]	Short time energy $^{L}$	Energy variation among frames
	[16, 45, 46]	MFCC(1–13)$^{L}$, ${\varDelta }$ MFCC(1–13)$^{L}$, ${\varDelta } {\varDelta }$MFCC(1–13)$^{L}$,	Subtle changes in the motion of articulators (Details [32, 42])
Transition	Present study	PTS	Ability to promptly interrupt/start vocal fold vibration
	Present study	ETS	Ability to promptly interrupt/start vocal fold vibration
	[34]	MFCC(1–12), ${\varDelta }$MFCC(1–12), ${\varDelta }{\varDelta }$MFCC(1–12),	Ability to promptly interrupt/start vocal fold vibration
	[34]	BBE(1–25)	Ability to promptly interrupt/start vocal fold vibration

The apex letter represents the classification between *LLf* and *HLf* subgroups

More in detail, we derived two classes of features for each of the voiced segments. The first group (Low Level features-LLf) encompasses parameters that are computed for each frame. The second one (High Level features-HLf) includes those features that are to be extracted from the entire signal (e.g. Detrended Fluctuation Analysis-DFA); indeed, their definition already embeds a comparison among contiguous frames.

After extracting such features, we evaluated four statistics per LLf (i.e. mean value of mean within a segment; standard deviation of mean within a segment; mean value of standard deviation within a segment; variation coefficient) and two statistics per HLf and transition features (i.e. mean value and standard deviation) on every recording. This allowed to perform the dimensionality reduction required without losing information on the temporal evolution of the signal. In particular, we remark that standard deviation yields information regarding the feature variability over time, which is known to be a crucial hallmark of PD patients’ voice impairment.

As for the specific features to be extracted, [10] emphasized the importance of differentiating the employed set according to the performed task (e.g. pronouncing sentences, isolated words, sustained vowels phonation). As discussed in section “Dataset”, the database used in this work includes isolated words speech recordings. A specific set of features with proven high correlation with this task is not available yet; hence, we extracted a total number of 126 features, aiming at investigating their correlation with the application at hand. This set encompasses features commonly involved in PD patients’ voice analysis (e.g. *F*0, MFCCs, Zero Crossing Rate-ZCR), in conjunction with general features used in vocal signal analysis (e.g. spectral flux, spectral centroid, spectral flatness) and others employed for the first time in this work (e.g. pitch transition slope-PTS, and energy transition slope-ETS). It is worth noting that pathological subjects exhibit an increased vibration aperiodicity [13], therefore specific algorithms are required to evaluate *F*0. In this work, we employed the Simple Inverse Filter Tracking (SIFT) algorithm, which guarantees the best trade-off between accuracy, noise robustness, and computational time when dealing with pathological voices [26].

**Fig. 3:**
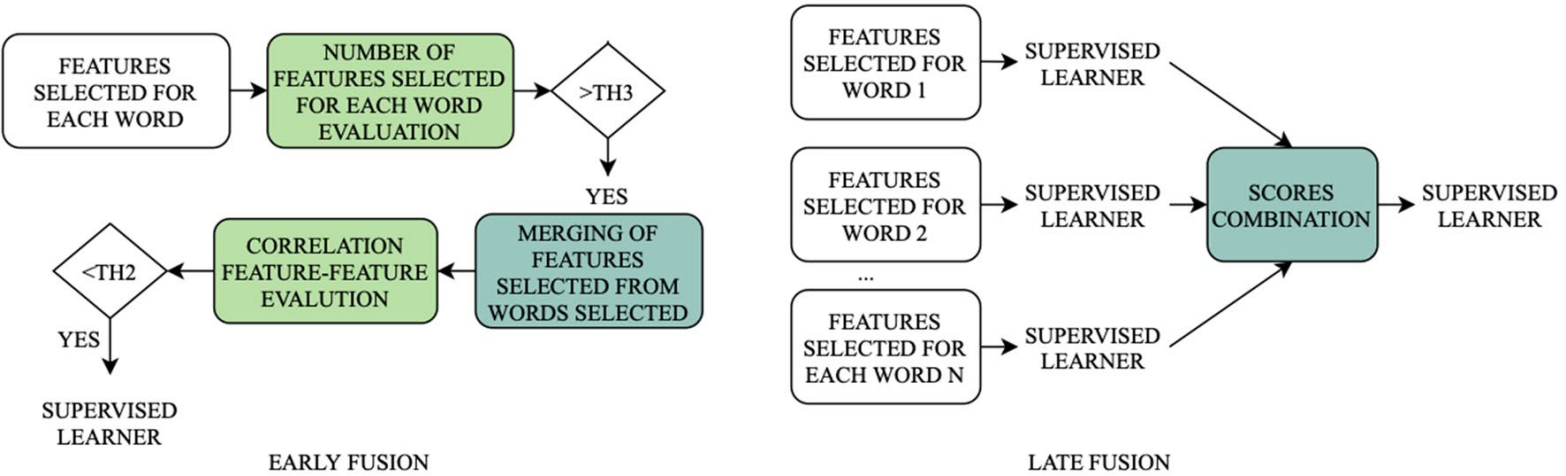
Comparison between early and late fusion approaches: in the first case, the features derived from each word are joined before performing the supervised learning; in the second one, separate scores are learned for each word, joined, and used as input of a second supervised learning step

As far as concerns PTS and ETS, these features aim at capturing articulation abnormalities in PD patients, particularly evident at the beginning or end of the voiced sound regions. This idea was originally introduced in [35] for speech signals and later validated in gait and handwriting [47]. In more detail, during the transition phase the phenomenon of *voicing leakage* commonly occurs [30]: the lack of coordination in the use of the source glottal leads to continuous vibration of the vocal folds even during the articulation of sounds, in lieu of an interruption of the phonation. This aspect is crucial in the automatic classification of PD patients by voice analysis [30, 34, 47]. Hence, we added these two novel parameters to others already addressed in [34] (MFCCs, BBEs), in order to capture as many facets of the alteration as possible. In particular, to analyze the voiced/unvoiced switch and vice-versa, we evaluated the pitch and energy contours in the transition regions using a first-order polynomial. Then, we employed the slope of the obtained curve as a measure of the alteration. In fact, we expect this curve to flatten in pathological voices when *F*0 and energy fail to change between voiced and unvoiced regions. Table 1 reports an overview of the features addressed in this paper, along with their classification into *LLf* and *HLf*, a brief description, and the reference to relevant papers. Given that different features exhibit different ranges, we applied the *Range* normalization (Eq. 2) to the whole feature set. Besides being a general good practice, this is particularly important if Euclidean distances are computed in the subsequent analysis (e.g. similarity measures).

2 $$\begin{aligned} f' = \frac{f - min(f)}{max(f) - min(f)} \end{aligned}$$

### Feature selection

In order to identify the smallest significant feature subset, we performed a tailored feature selection on the PC-GITA database. This procedure is meant to select the most significant (i.e. those with high feature-target correlation) and non-redundant features (i.e. those with low inter-feature correlation). To avoid model overfitting on training data, we implemented a correlation-based approach. In fact, the correlation coefficient is weakly affected by a single data-point and it is mostly influenced by the gross data distribution. Furthermore, to avoid weak generalization capability, possibly due to feature selection performed on all the available data, we randomly split the database into two subsets: 70% to be used during the training/validation phase and 30% to be used as test set. The two sub-groups were chosen in such a way as to guarantee speaker independence (i.e. all words of the same speaker are either in train or test, but not distributed between the two subsets). First, we computed the Pearson’s correlation *r* between features and target ($r_{fo}$), investigating its absolute value for each feature. The objective is to identify features having a strong correlation with the output (i.e. $r_{fo}$ greater than a threshold-$th_1$). To select the threshold properly, we performed a tuning procedure, within the 70% of data selected, based on the misclassification error minimization in 10-fold cross-validation, using a quadratic SVM. We are aware that this step could introduce a bias; however, given the low number of parameters to optimize, the bias is minimal. More in detail, we tuned $th_1$ from 0.3 to 0.6 with steps of 0.1. At this stage, considering that the two databases used in this work encompass 25 isolated words for each subject, we compared the two approaches, aiming at identifying the best model capable of capturing as much information as possible from several utterances pronounced by the same subject. To this end we considered two types of fusion schemes, namely *early fusion* and *late fusion*. The former performs the fusion in the feature space, while the latter fuses features in the semantic space [44]. Figure 3 shows a schematic of the differences between the two approaches. As for *late fusion*, it consists in implementing a classifier for each word, then using the output of such models to feed a further classifier, obtaining the final output. To this end, we employed features selected from each word as input of 25 supervised classifiers (one for each utterance), then we converted the output using Platt’s method [44] to acquire a measure in the form of a probability score. Then, we merged the probabilistic output scores and used them as input of a second classification layer. As for *early fusion*, it consists in the apriori selection of the most significant words and then merging the features from such words to create the final feature set. To this end, we performed an additional analysis to select the most significant utterances, keeping those characterized by a number of selected features per word $f_{w}$ higher than $th_3$. We tuned $th_3$ from 1 to 80 with steps of 5. After merging all the features from the selected word, we computed the correlation coefficient between feature pairs ($r_{ff}$). Then we deleted redundant features, i.e. those showing a $r_{ff} > th_2 \cdot r_{fo}$. We tuned $th_2$ from 0 to 50% with steps of 5%, choosing the value minimizing the misclassification rate in 10-fold cross-validation. The entire process is reported in Algorithm 1.
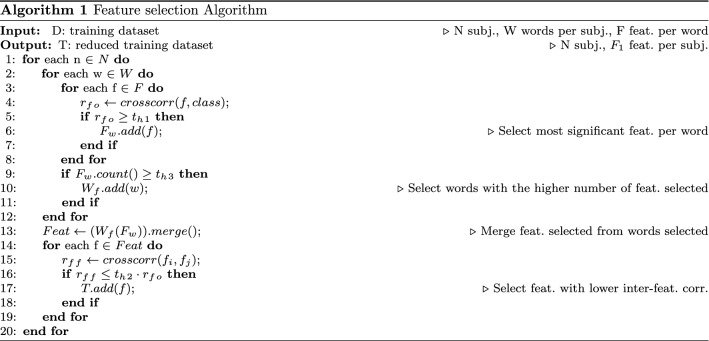


We performed feature selection on three different subsets of features:

- Set 1: Only features extracted from the entire signal are employed;
- Set 2: Features extracted from the entire signal and features extracted from the voiced segments are employed;
- Set 3: Features extracted from the entire signal and features extracted from the voiced and transitions segments are employed.

It is worth noting that, as far as concerns set 1, no feature selection was performed due to the small number of features belonging to this set (i.e. only 6 parameters).

### Classification

In this study, we decided to employ a quadratic SVM to perform the initial supervised learning steps due to the high generalization capability of the algorithm [8] and its widespread use in PD patients voice classification [22, 29, 39]. More in detail, as mentioned in section “Feature selection”, we first compared the classification performance obtained using early and late fusion to assess how the composition of the feature set affects the classification results. To this end, we implemented a quadratic SVM model and performed 10-fold cross-validation on the training set using the different feature sets described in section “Feature selection”. As the dataset is balanced (i.e. the cardinality was the same in every class), we considered accuracy a good metric for performance evaluation.

Once identified the best fusion scheme, we compared the quadratic SVM with other classifiers to study whether different algorithms can lead to better classification accuracy. In more detail, we compared the quadratic SVM to the k-Nearest Neighbour (kNN), naive bayes (NB), decision tree (DT), bagged trees ensemble, and subspace discriminant ensemble. After identifying the best classifier, we performed an optimization step by mean of a grid search approach to evaluate the best hyper-parameters for the model. More specifically, we considered four distance metrics (i.e. euclidean, city block, Minkowski, and Chebyshev) and k values ranging from 2 to N/2 (with N equal to the number of samples in the training test). In the case of equal optimal accuracy, we preferred lower k-values to reduce the computational burden of our model. In virtue of the random splitting procedure employed, we considered the average accuracy on five iterations as a good metric for the optimization procedure. Finally, to provide a comprehensive analysis of our model’s performance, together with the classification accuracy, we examined the time complexity of the algorithm. More in detail, we computed the computational time required to run the classification algorithm when varying the input size, the number of words employed, and the number of features.

We ran all the experiments on a MacBook Pro with a 64-bit operating system, a 2.7GHz Intel Core i5 processor, and 8GB RAM.

## Results and discussion

In this section, we present and discuss the results of the current study with the aim of selecting the most effective vocal features to be extracted from isolated words speech samples pronounced by PD patients. In more detail, we compare the performance of early and late fusion approaches, and the corresponding computational time; we report the performance of different ML models in discriminating PD subjects from HC; we provide a comprehensive analysis of the time complexity of the proposed algorithm; we report and discuss the most meaningful features and words, and finally we compare the performance achieved in the present study with that reported in similar works.

### Early fusion vs late fusion

**Fig. 4:**
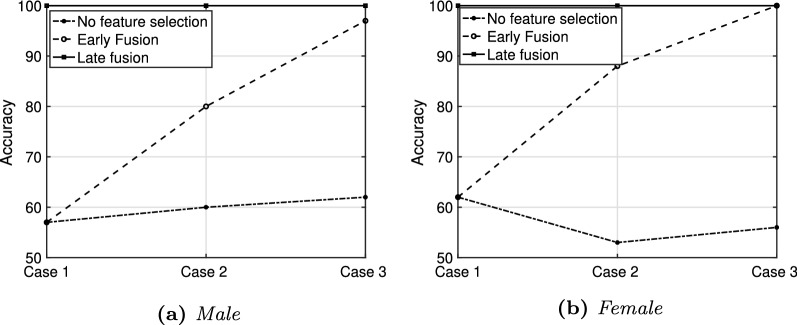
Fusion scheme and feature subset analysis. Performance for the first randomly selected subset

In Fig. 4 we report a comparison between early and late fusion approaches for the three different feature sets. Results are expressed in terms of accuracy in a *10-fold* cross-validation, using a non-optimized quadratic SVM classifier and employing the first randomly selected training set. For the sake of comparison, the results achieved merging all the features extracted from each word without performing any feature selection procedure are also shown. From Fig. 4 it can be appreciated that increasing the dimension of the feature set by adding more specific features (i.e. voiced segments and transition regions), enhances the performance of the model in the cross-validation phase. In contrast to early fusion and no-feature-selection configurations, the late fusion scheme exhibits a flat course with optimal performance, suggesting possible overfitting of such configuration. Nevertheless, it is evident that the best system configurations are early fusion employing the entire feature set, and late fusion regardless of the used feature set. To provide an insight into the generalization capability of each of the best configurations, we ran tests on 30% of PC-GITA extracted from the initial dataset before selecting the features and optimizing the model. While the late fusion results were not satisfactory, the case3-early fusion configuration showed an accuracy of 82% (average value over 5 iterations for male and female groups), which demonstrates the good generalization capability of the system.

Furthermore, we assessed the computational time employed by each feature set-fusion scheme configuration. We computed time from feature selection to classification and compared it among different models.

Table 2 shows the time employed for processing and classifying, proving that the computation burden is far smaller for the early fusion configuration. The execution time reported in Table 2 is defined as the time required to select relevant features and words, and test on a new single subject.

**Table 2:** Execution time of the three most proficient algorithms

Model	Computational time (s)
Case 1: Late fusion	3.37
Case 2: Late fusion	4.19
Case 3: Late fusion	6.23
Case 3: Early fusion	0.065

Mean values reported between male and female subjects

### Classification models

After identifying the best fusion scheme, we investigated if different classification algorithms and their involved meta-parameters led to better performance. Table 3 reports the results of the comparison expressed as average accuracy on 5 iterations obtained using non-optimized classification algorithms. It is worth noting that we decided not to use more complex methods, such as artificial neural networks, due to the scarce numerosity of training data.

**Table 3:** Performance comparison among 6 classifiers

Classifier	Male		Female	
	Validation set	Test set	Validation set	Test set
SVM	96% ± 3.22	74% ± 18.95	98% ± 2.46	90% ± 7.12
DT	95% ± 4.46	64% ± 17.34	100% ± 0	65% ± 19.56
NB	73% ± 41.10	50% ± 28.36	92% ± 5.65	77% ± 22.36
kNN	96% ± 2.46	74% ± 15.56	99% ± 1.61	97% ± 3.42
Ensemble bagged trees	92% ± 5.05	60% ± 19.56	96% ± 1.31	56% ± 0
Ensemble subspace discriminant	94% ± 5.26	71% ± 16.29	99% ± 1.31	96% ± 3.42

The results report the validation (10-fold applied to 70% of PC-GITA) and test set (30%PC-GITA) accuracy averaged over 5 iterations

**Table 4:** Performance comparison among validation set (10-fold applied to 70% of PC-GITA), test set (30%PC-GITA) over 5 iterations for male and female groups

	Iter.	Validation set				Test set				kNN optimal parameters
		Acc.	Sens.	Spec.	AUC	Acc.	Sens.	Spec.	AUC	
**Female**	1	100%	100%	100%	1	100%	100%	100%	1	Distance = cityblock K = 3
	2	100%	100%	100%	1	94%	100%	87%	0.94	
	3	100%	100%	100%	1	100%	100%	100%	1	
	4	97%	100%	94%	1	100%	100%	100%	1	
	5	100%	100%	100%	1	94%	87%	100%	0.94	
	**Mean**	99.4%	100%	98.8%	1	97.6%	97.4%	97.4%	0.98	
Male	1	100%	100%	100%	1	100%	100%	100%	1	distance = cityblock K = 6
	2	100%	100%	100%	1	75%	63%	87%	0.75	
	3	97%	94%	100%	0.97	87%	75%	100%	0.88	
	4	100%	100%	100%	1	100%	100%	100%	1	
	5	100%	100%	100%	1	94%	88%	100%	0.94	
	**Mean**	99.4%	98.8%	100%	0.99	91.2%	85.2%	97.4%	0.91	

The model optimal hyper-parameters are reported

As can be seen from the Table 3, kNN enhances a slight improvement of the performance both in validation and test set. Moreover, a smaller standard deviation indicates more consistent results across random splits. Then, we evaluated the computational time required to select relevant features and words, and test on a new single subject using the kNN model. This value was equal to 0.047s (mean value for male and female subjects), thus confirming the improvement achievable with the kNN algorithm. As for the optimized model parameters, city block distance and k equal to 6 for males and 3 for females led to the best performance.

Table 5 reports the optimal parameters (found on PC-GITA according to the procedure described in section “Feature selection”) used for the final test.

**Table 5:** Set of feature selection parameters employed for the final test

Parameter	Male value	Female value
th1	0.5	0.5
th2	0.1	0.1
th3	10	30

In order to further investigate the possible presence of overfitting, we tested the final model on the validation set and the test set, i.e. 30% of the training set. Since the splitting procedure is random, we report in Table 4 the performance obtained by running the algorithm five times.

It can be observed that the model obtained optimal correct classification rate in both validation and test set, although the selection of different inputs has a strong influence on the algorithm performance. This is particularly evident in the male group, in which the classification accuracy varies from 75 to 100%. This influence is less evident in the female group, in which the classification accuracy remains equal to 100% in 3 of the 5 subsets analyzed. Given that we observed higher standard deviation and lower performance in the male population with most of the models tested (as reported in Fig. 4 and Table 3), we may assume that this is mainly due to the dataset composition itself. Also, to analyze how the recording condition may affect the performance of the implemented model, we performed further tests on the additional dataset, described in section “Dataset” We achieved an average accuracy over 5 iterations equal to 60% and 62% for male and female subgroups, respectively. A general performance reduction is evident in the additional dataset, especially in the male group. Given that the analysis conducted on the test set resulted in the absence of strong overfitting, we can assume that this reduction is mainly attributable to the different recording conditions which characterize samples in the new dataset (section “Dataset”). Nevertheless, further analysis, such as the introduction of new subjects into the database, will be conducted to assess the robustness of the current algorithm through a more homogeneous training set.

### Time complexity

Together with the classification accuracy, we studied the time complexity of the algorithm. The pseudo-code for feature selection (section “Feature selection”) is reported in Algorithm 1. For this analysis, we assume that the number of training subjects is *N*, the number of words per subject is *W*, the number of initial features per word is *F*, and the final number of features selected is $F_1$.

**Fig. 5:**
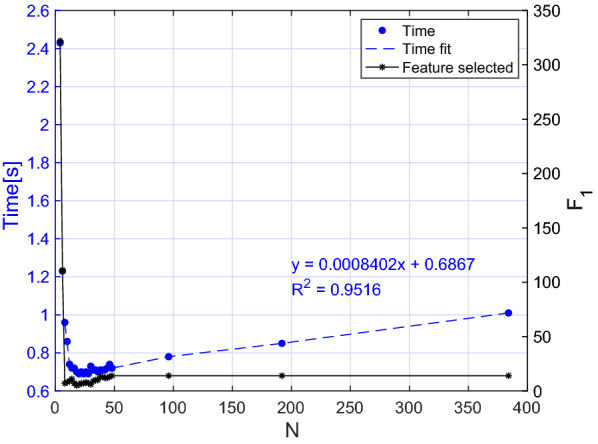
Execution time of the algorithm with different numbers of inputs. The final number of features selected is also reported

**Fig. 6:**
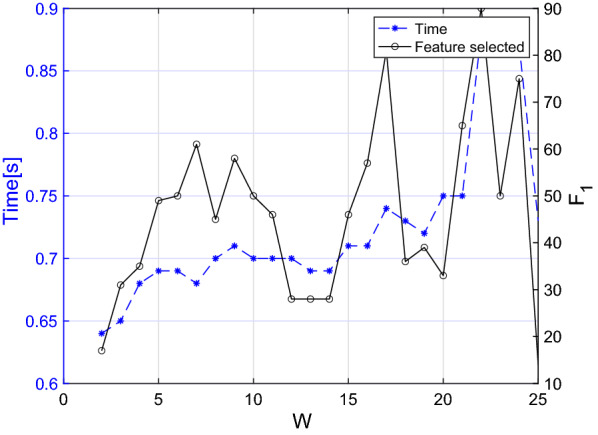
Execution time of the algorithm with different numbers of words. The final number of features selected is also reported

**Fig. 7:**
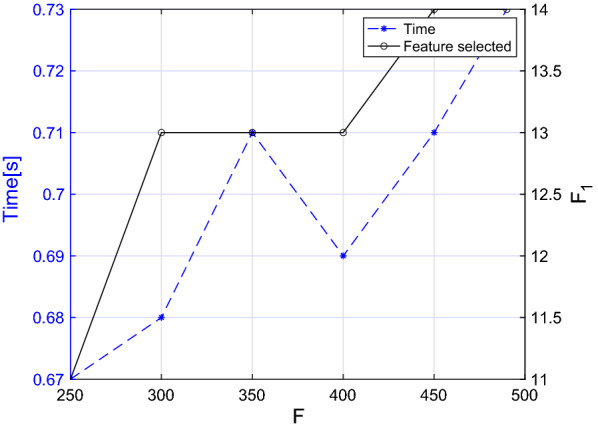
Execution time of the algorithm with different numbers of input features. The final number of features selected is also reported

More in detail, in Algorithm 1 the selection of the most significant features per words and the selection of the words with the higher number of features selected takes *O*(*nfw*). As for the selection of the features with the lower inter-features correlation, it takes $O(nf_1^2)$ since it includes the evaluation of the Pearson correlation coefficient between couple of features. In the worst-case scenario (i.e. when all the features and the words are selected), $f_1$ is equal to $f \cdot w$. Thereafter, we can conclude that the feature selection algorithm takes at most $O(nf^2w^2)$. As far as concerns the classification algorithm, the kNN algorithm takes *O*(*log*(*n*)) in Matlab environment [9]; hence, we can assume that the worst-case scenario time complexity of the overall algorithm is $O(nf^2w^2)$. To verify this theoretical result, we ran the algorithm several times with different numbers of training inputs, features, and words. Moreover, we estimated the value of $f_1$ for each iteration. For the sake of brevity, given that we applied the same process to both female and male dataset, we present the analysis for the former group. The results are showed in Figs. 5, 6, and 7. We consider the execution time as the time required for feature selection, training, and testing on a new subject. Moreover, to provide more stable results, we ran each experiment 5 times on a random extracted subset and reported the average time value.

In Fig. 5 we report the execution time versus *N*. It is worth noting that to perform a realistic analysis despite the scarce numerosity of the current dataset, after tuning *N* from 2 to 49, we performed additional measures on a simulated larger dataset obtained by including the same samples multiple times. As can be observed in Fig. 5, the number of features selected is almost independent of N, but if the number of training samples excessively decreases (i.e. less than 6 subjects per group), a higher number of features is selected. This is because our selection procedure relies on the Pearson correlation coefficient, whose value is inversely proportional to the variance of the training group. Thereafter if the number of inputs is excessively small, a large number of features is associated to a higher correlation coefficient. However, if we consider in this analysis only the region where our model is stable (i.e. more than 6 subjects per group), the regression line of the curve shows as expected a linear trend ($R^2 = 0.9516$).

As far as concerns Figs. 6 and 7 , in the former we reported the execution time while tuning the number of words from 1 to 25, while in the latter we progressively decreased the number of parameters until our feature selection algorithm was still applicable. In fact, if the number of initial characteristics for each word excessively decreases, the set of words having a number of features higher than the threshold specified in Table 5 is empty. From both figures it becomes evident that the computational time is generally increasing as W ($r = 0.77, P<0.001$) and F ($r = 0.88, P = 0.020$) increase although it is not possible to clearly see the nature of this relationship. In fact, even if the curve trend is increasing overall, the punctual value also depends on $F_1$, whose value strongly depends on which words and features were used for the specific iteration.

### Post-hoc analysis of the model and comparison with similar studies

In Table 6 we report the most significant words and features for male and female subgroups resulted from the post-hoc analysis of the selected models. As can be seen from the table, most of the features have been selected from the transition regions, confirming their potential in PD speech analysis. Among these, PTS and ETS were selected for the female and male group, leading to the assumption that these new features could be representative of the pathological condition.

Table 7 provides a comprehensive evaluation of the system performance and a comparison with similar studies employing isolated words contained in the PC-GITA corpus. The comparative analysis takes into account the best validation results reported in [36] (10-fold cross-validation), in [22](LOSO validation), and in [49](5-fold validation). It is worth noting that we excluded [37] from this comparison. In fact, although the work employed the PC-GITA corpus, the model was optimized on the test set, yielding too optimistic results as reported by the authors themselves and mentioned in section “Related work”. From Table 7 it turns clear that performance metrics of the proposed algorithm outperform those of the studies under comparison. On the other hand, it does not encompass a large cohort of PD patients;therefore, future developments will include a larger population. Furthermore, the non-negligible variance among performance achieved during the training of the algorithm with different subsets of patients will be further investigated by enlarging the current database.

As for the types of features employed, we emphasize the advisability of designing gender-specific software. In fact, the feature selection process described in section “Feature selection” yielded two different subsets of significant words and features for the two gender subgroups. Furthermore, the presence of specific training sets leads to slightly diverse classification algorithms, due to the different values yielded by the optimization process described in section “Classification”.

**Table 6:** Most significant words and features for male and female subgroups resulted from the post-hoc analysis of the selected models

	Words selected	Feature name	Region
F	Clavo, Crema, Globo, Name	Roll off point	Voiced
		MFCC, BBE, ${\varDelta }{\varDelta }$MFCC	Onset
		PTS, ETS, MFCC, BBE, ${\varDelta }{\varDelta }$MFCC	Offset
M	Bodega, Braso, Globo, Llueve, Name, Presa, Viaje	MFCC, BBE, ${\varDelta }{\varDelta }$MFCC	Onset
		PTS, MFCC,BBE	Offset

*F* female, *M* male

**Table 7:** Performance comparison with the best results of similar studies employing the PC-GITA database and focusing on the isolated word repetition task

Author	[36]	[22]	[49]	Present study
Year	2015	2020	2020	2020
Model	SVM	SVM	CNN	kNN
Sensibility	94%	n.r.	n.r.	99.4%
Specificity	90%	n.r.	n.r	99.4%
Accuracy	92%	91%	77%	99.4%
F1-score	n.r.	0.83	n.r.	0.99

*n.r.* not reported. For the present study mean values between male and female subgroups averaged over 5 repetitions are reported

## Conclusions and future work

In this paper, we addressed the language impairment of PD patients, based on the analysis of recordings of isolated words. We chiefly focused our effort on feature extraction and selection to devise a lightweight but very performing ML model for classification. In fact, once identified the best feature subset, the feature extraction and subsequent classification tasks are very computationally efficient. This work confirmed the possibility of a speech-based PD classification, suggesting new promising methodologies for vocal feature analysis. Furthermore, the usage of features extracted from common words gives rise to a new perspective on passive speech-based monitoring of PD patients. Specifically, given the high precision reached by our algorithm, it may be employed in the home monitoring of motor fluctuations in PD subjects, as well as a decision support system in early PD diagnosis. On the other hand, given the reduced size of the dataset employed in this study, our methods and results require further validation with a much larger cohort of subjects. We intend to check whether the subject’s native language can influence the classification results and, if so, to what extent. Besides collecting additional speech data from PD patients, we also plan to employ precious clinical information (e.g. H&Y stage, UPDRS, Mini-Mental State Test). We plan to perform data acquisition several times on the same patients, both in ON and OFF clinical conditions. This would allow to compute the test-retest reliability of the system, as well as understanding whether the system is capable of detecting clinical conditions. Finally, this study is part of a larger PD monitoring study [5], involving the implementation of an *electronic diary* for PD patients, which will combine the assessment of the main PD motor symptoms (e.g. bradykinesia freezing of gait, postural instability) as well as sleep disturbances.

## Abbreviations

PD: Parkinson’s disease
HC: Healthy controls
MDS: Movement Disorder Society
UPDRS: Unified Parkinson’s Disease Rating Scale
MFCC: Mel-Frequency Cepstral Coefficients
GMM: Gaussian Mixture Model
IEDCC: Instantaneous energy deviation coefficients
NHR: Noise to harmonic ratio
SVM: Support vector machine
SIFT: Simple inverse filter tracking
CNN: Convolutional neural network
ZCR: Zero Crossing Rate
LPF: Low-pass filter
ML: Machine learning
LOSO: Leave one subject out
LPC: Linear prediction coefficients
BBE: Bark band energy
DFA: Detrended fluctuation analysis
PTS: Pitch transition slope
ETS: Energy transition slope
kNN: K-Nearest neighbors
DT: Decision tree
NB: Naive Bayes
